# Clinical Researches upon Auscultation of the Head

**Published:** 1862-12

**Authors:** Henry Roger

**Affiliations:** Prof. Fac. Med., Paris


### CLINICAL RESEARCHES UPON AUSCULTATION OF THE HEAD.

By HENRY ROGER, M. D., Prof. Fac. Med. Paris.

It is well known that the first application of the discovery of Laennec to the diagnosis of diseases of the brain was made by Dr. Fisher, of Boston. In 1832 this physician communicated to a learned society a memoir upon the *cephalic bellows sound*, and transmitted his interesting researches to the *American Journal of Medical Science*, Aug. 1838. "Auscultation," said Dr. Fisher, "may be as useful in the symptomatology of the affections of the brain as it is in those of the chest, and may furnish a pathognomonic sign of these diseases."

After having traced the rules of cerebral auscultation, and indicated the normal sounds which reach the ear when applied to the head, and having distinguished those which come from the nasal fossæ, and which belong to respiration and phonation, as well as those which proceed from deglutition and from the head itself; after having distinguished all these from abnormal sounds, Dr. Fisher considers the *cephalic souffle* in diseases of encephalon. He mentions, among other affections of which an abnormal bruit is one of the symptoms, *chronic hydrocephalus*, *cerebral congestion*, either simple or resulting from disordered dentition or hooping-cough, *accute inflammation of the encephalon or its membranes*, *abscess* of the brain and *induration* of that organ.

Dr. Whitney went even further than his colleague in his opinions with regard to the symptomatological value of the cephalic souffle (*American Journal of Medical Science*, Oct. 1843). To the affections mentioned by Dr. Fisher—cerebral congestion, meningitis, &c., in which the stethoscopic phenomena were present, he adds scirrhous transformation of the cerebellum, and mechanical compression of the brain. He says, moreover, that he perceived a *cerebral œgophong* in hydrocephalus, a bruit similar to the *catarrhal fremitus* in aneurism of the arteries at the base of the brain, and finally he points (and with reason, in certain cases) to the existence of a *cephalic souffle* in *anœmia of the brain* and also in *chlorosis*.

Notwithstanding the labors of these two American authorities, each of whom confirmed the observations made by the other, and in spite of results which generally agree, and which seem to have the testimony of numbers—for the facts in their memoirs count by hundreds—cerebral auscultation, which promised to throw an unexpected light upon a very obscure point in pathology, met with but little favor. The European medical journals confined themselves to incomplete analyses and short extracts; while but few practitioners thought it their duty to repeat the experiments necessary for a criticism of the facts which had been stated by our American brethren, as if they mistrusted a discovery coming from such a distance.

In the year 1841, in the first edition of the *Traite Pratique d'Auscultation*, mention was made of the observations of Drs. Fisher and Whitney; but the experiments to which M. Barth and I devoted ourselves for testing their truth, though certainly not very numerous, only gave us negative results. After having searched in vain to discover a cephalic souffle in certain cerebral affections, and having been unable, in twelve or fifteen cases of meningitis, to hear any such, while the American doctors declared it to be present in all; strengthened besides in our scepticism by the silence maintained by MM. Andral and Bouillaud upon auscultation of the head, and by the equally negative observations of MM. Fournet and Vernois, in nine or ten cases of meningitis, and of Professor Piorry,* we thought it right at that time, and in subsequent editions of our work, to express doubts of this new application of the stethoscope, and were led to dispute, if not the reality, at least the importance of auscultation of the brain.

Still I always regretted this summary condemnation of the undoubtedly conscientious labors of two professional brethren. That results stated in America as certain should be absolutely and generally denied in Europe seemed not a little extraordinary; and we must either suspect the mode in which the physicians on the other side of the Atlantic made their obsertions, or confess that in this hemisphere we were deaf to sounds distinctly heard in the other. Shall we say with Harvey's adversary, the old Venetian physician, "*quem nos surdastri audire non possumus*"—" in Europe people are deaf; they only hear that in America '*tantum modo in America exaudi-tur.*' "

As the only means of deciding the question was by making

* Traite de pathologie iatrique, tom. viii, p. 353.

more minute clinical observations, I devoted myself patiently to this difficult task, and began afresh the study of cerebral auscultation. My researches were almost exclusively among children,* because it is chiefly among them, when at the breast, that Drs. Fisher and Whitney said they heard the stethoscopic phenomena, the discovery of which they announced; and because the difficulty of diagnosing diseases of the brain is greater in childhood than in later life, a greater interest belongs to the progress which symptomatology would make in this department. I may add in passing that it is only among children, whether subject or not to cerebral disease, that auscultation of the brain is likely to be of use; for beyond a certain age, or beyond a certain period in childhood, the most practised ear applied to the cranium cannot detect morbid cephalic bruits.

Since I began collecting the observations which form the subject of this memoir, several authors have been similarly engaged in cerebral auscultation, among others MM. Rilliet and Barthez, and M. Hennig, head of the Children's Hospital at Leipsic. The first, in their admirable work (2d edit., vol. ii. pp. 158–9), make some remarks on the cephalic souffle in cases of hydrocephalus and in rachitis; I shall have occasion to quote the passage where they speak of this bruit under the head of the diagnosis of these affections. The German author has written a monograph upon this subject, and in a pamphlet of not less than thirty pages (*Archiv fur physiologische Heilkunde.* Stuttgart, Aout 1856) he reproduces, with annotations and additions, the inaugural thesis of Dr. Wirthgen (*De strepitue qui in capite auscultando auditur*, Leipsic, 1855), which indeed appears to owe its birth to him, and in which he successively studies the normal and abnormal bruits discovered by cerebral auscultation, the seat of those bruits, the age at which they are heard, their physiology and pathology, and especially the mechanism of their formation, and from these he deduces the results which the physician may expect to find in practice. I had completely devoted myself to the study before I became acquainted with the researches of M. Hennig, and we shall see, by the details of my observations, how far my conclusions agree with his.

Perhaps the admirable work of the Leipsic physician is faulty from a want of method and perspicuity, of which French

* I have never been able to detect any cephalic bruit among adults, either in apoplectic patients, or young chloro-anæmic females.

readers complain greatly in the writings of the learned German author. M. Hennig does not give with sufficient clearness the details from which he drew his deductions, nor his mode of proceeding in collecting facts; the number of cases from which he decides (and they do not appear to be very numerous) are not indicated with precision; all this necessarily diminishes the value of his statements.

In order that I may not incur the reproach just cast upon the German author, I may say that the conclusions which are recorded in this paper were drawn from an examination and comparison of a very considerable number of observations, their total being nearly 300; at first they were gathered from cases selected from among children whom I thought were affected with cerebral disease, or were the subjects of rickets; then (having determined the existence of a cephalic souffle among children of healthy appearance) I afterwards examined all cases indiscriminately, whether in the wards of the Hospice des Nouveaux Nes, the Hopital des Enfants, or in private practice.

*Plan of the Memoir.*—Having made these preliminary observations, we come now to the study of facts, and after having traced out the *rules* which facilitate auscultation of the head, I shall point out what *physiological bruits* the ear discovers when applied to the cranium; and in a third chapter, the longest and most important, *morbid bruits* will be specially considered.

SECTION 1. *Rules for Cerebral Auscultation.*—When a patient is submitted to stethoscopic examination the most convenient position is the horizontal, with the head placed upon a pillow slightly raised; this may be inclined to different sides, according as it is desirable to examine the occipital, frontal, or temporal regions, etc.

In examining an infant at the breast, it should be seated on the knee of its mother or nurse, and the head should, if possible, be held steadily, either by the hands placed softly on either side, or by leaning it against the mother's breast. If the patient be in a comatose condition from some cerebral affection, auscultation will be easily performed. It becomes difficult when the child cries or moves about, which is usually the case; we must then try to quiet it by suckling and auscult it while it is at the breast, the bruit of deglutition being no obstacle to the recognition of morbid cephalic bruits.

If the examination be impossible while awake, it must be abandoned till an attempt can be made in quieter moments, or we may make the attempt during sleep, which is generally sufficiently profound not to be disturbed by the examination of a physician.

The auscultation may be either *direct* or *indirect.* Dr. Fisher found the latter the most convenient, the ear adapting itself exactly to the rounded or projecting surface of the cranium. Care must be taken to prevent the rustling of the hair; also, as a matter of cleanliness, the child's head should be previously covered with a napkin, which may serve at the same time to keep it more firm. Auscultation, practised thus directly, possesses some advantages; whatever may be the cries or agitation of the little patient, one always finds, in applying the ear quickly and somewhat firmly to the middle of the anterior fontanelle, that we catch, so to say, the sound of a cephalic souffle. But when the child is asleep, or when it is willing to remain quiet, mediate auscultation is preferable; it allows the detection of the bruits with greater precision and exactness, and we may also isolate them, so that those which are natural are not confounded with those which are abnormal.

For this purpose the ordinary stethoscope, which has been generally substituted for the primitive cylinder of Laennec,* answers perfectly; held as a pen in writing, it should be applied over the different regions of the cranium, and be kept fixed in the same way as for auscultations of the vessels of the neck. M. Hennig recommends the employment of a flexible stethoscope, twenty-three centimetres long, having for its middle portion a tube of vulcanized India-rubber. This stethoscope is certainly preferable, for with it we can easily follow the movements of the child during the examination.

On what regions of the head should the instrument be applied in order the better to detect the physical phenomena? If Drs. Fisher and Whitney are correct, the bruit of the cephalic souffle is so evident that it suffices to apply the ear to any parts of the cranium in order to discover it immediately; but our experience does not bear out this assertion. It may be true that the bruits of nasal respiration, and of deglutition, are perceptible over nearly the whole surface of the head, but this is certainly not the case with the genuine cephalic souffle, which we have most frequently heard in the region of the anterior fontanelle, and still more plainly when we auscult over the fontanelle itself. It gradually diminishes in intensity the further we remove from that point, which ap-

* Traite Pratique d'Auscultation, cinquieme edition, p. 19.

pears to be the center whence the bruit starts; then gradually becoming more feeble along the parietal or frontal sutures, it is completely lost further away, the finest ear failing to discover any sound over the lateral surface of the cranium and the occipital protuberances.

The physician who cannot count for long docility of his little patient would do well, if he wishes to detect the cephalic bruit, at once to place his stethoscope on the summit of the cranium over the depression corresponding to the anterior fontanelle; it is there, as we have said, that the abnormal bruit exists in its greatest intensity, and the examiner, if he does not detect it at that point, may dispense with any further investigation.

SECTION II. *Physiological Bruits.*—When we practice auscultation on the top of the head of a healthy subject, different bruits are heard.*

I. One is produced by the circulation of the air in the nasal fossæ; this is the *cephalic bruit of respiration* mentioned by Dr. Fisher; it is very intense and harsh, and analogous to the laryngeal respiratory murmur; it coincides with the respiratory movements, increasing in strength and frequency when these are accelerated.

These characters are sufficient to distinguish it from other cephalic bruits; but in some cases of extreme dyspnœa the nasal respiratory bruit is so frequent and quick that it might easily be mistaken for the cerebral vascular souffle of which we have just spoken, and the two phenomena might be confounded were it not for the close relation which the first bears to the movements of the thorax.

When the pituitary membrane is thickened or there is much secretion of mucus, the respiratory cephalic bruit is transformed into a sibilant rhonchus, or into a kind of coarse crepitation; the same physical conditions of the mucous membrane exist as in bronchitis, and consequently the same rales are heard.

Nor is this all; in affections of the larynx, the bruits caused by the passage of the air along the laryngo-tracheal tube are drier and more or less prolonged, extending above into the nasal fossæ as well as into the bronchial tubes below, and the rhonchus or laryngeal hissing (similar to the bronchial rales)

* In treating here of *auscultation of the head,* I shall not consider *aneurisms* of the larger arteries of the brain, nor *diseases of the ear* (see the work of M. Gendrin and an admirable article by M. Menier, in the *Traite pratique d' Auscultation,* 5th edit. p. 562.)

everywhere heard is more perceptible in auscultation of the head than it is in that of the chest.

With regard to the rales formed in the lungs, these are not heard from so great a distance, and they escape stethoscopic examination when practised on the head; in the very exceptional case of a young phthisical girl I was able to detect, by cerebral auscultation, a loud gurgling which was produced in a cavity in the subclavicular region.

II. If the child speaks or cries the *vocal sounds* are heard very distinctly, and the ear, when applied to different parts of the cranium, perceives a very remarkable resonance; this *cephalic resonance* may be compared to that which is heard so loudly in the larynx, it has nasal timbre which varies in different individuals, it is always very piercing, the voice seeming to proceed from the cranium.

III. Placing the stethoscope on the head, another peculiar bruit may be heard, that of deglutition; in new-born infants there is also that of suction. This bruit is so singular and so readily recognized that there is no need to describe it. We hear it more distinctly by direct than indirect auscultation, and in cases where the nurse appears to have but little milk, and when consequently the child makes frequent efforts at suction without our being able to decide the fact of its having swallowed anything, we may be able to determine this by cerebral auscultation, the bruit of deglutition comes very strongly upon the ear, and we are therefore better able to appreciate the quantity of milk supplied and swallowed.

Such are the *extrinsic bruits* revealed by cerebral auscultation; moreover, it is not impossible to a practised ear to hear in some subjects, especially in cases of palpitation, the two sounds of the heart propagated from the solid walls of the cardiac region up to the head. In forty-two healthy infants five times I was able with attention and patience to perceive the tic tac of the heart in ausculting over the cranium. In another case, I have been able to hear a strong bellows-sound originating in the left auriculo-ventricular orifice; but this last case was exceptional. More frequently the cardiac sounds themselves escape observation in auscultation of the brain; and we may, for greater simplicity in studying the subject, omit the *cardiac cephalic bruit*, at least as it has been described by Drs. Fisher and Whitney.

With much greater reason we shall contest the possibility of recognizing with the ear the modifications which so-called cerebral diseases create: for example, Dr. Fisher says he is able to discover in almost every case (thanks to his aptitude for cerebral auscultation), the impulsive character of the pulsation which he supposed to belong to apoplexy. These, however, are minutiæ of such exceptional cases (*rara non sunt artis*), and tend only to embarrass without enriching semeiology.

IV. Are we, then, to include in the physiology of the subject an *intrinsic bruit*, the *cephalic souffle*, an essentially pathological phenomenon?

On this point, those authors who have specially directed their attention to auscultation of the brain are far from being agreed. With the American observers, the souffle is always abnormal, it is never a healthy phenomenon. Dr. Fisher expressly says, "it depends always upon some cerebral affection." Dr. Whitney equally insists upon the proposition, that auscultation never reveals anything like a cephalic souffle in the normal condition.

M. Hennig, on the other hand, believes that the souffle belongs rather to a state of health;* and, further, this bruit, which according to the American doctors would be much more manifest as the cerebral affection became more marked, would, according to the Leipsic physician, sometimes disappear in sick children, to reappear with returning health.

If we seek among our own facts whether the American or German authors are right, the first declaring that in the normal state the cephalic souffle is never heard, and the second being of opinion that it is always heard; we incline to believe that both are equally wrong, and that these two exclusive opinions are alike to be condemned.—*London Med. Rev.—American Journal.*

---

* So we gather from several confused passages in his Memoir where cephalic bruits are considered. "We have heard these bruits (M. H. does not say which) on the head from the twentieth week to the sixth year.——These bruits have been present in healthy children at the twenty third week." (*Loco cit.* p. 413.) "The souffle or hissing which is present in *healthy* children. (*Ibid.* p. 415.) "These bruits are sometimes wanting in sick children, but reappear with convalescence." (*Ibid.* p. 413.)

